# Uncommon liver tumors: Case report and literature review

**DOI:** 10.1097/MD.0000000000004952

**Published:** 2016-09-30

**Authors:** Chia-Hung Wu, Nai-Chi Chiu, Yi-Chen Yeh, Yu Kuo, Sz-Shian Yu, Ching-Yao Weng, Chien-An Liu, Yi-Hong Chou, Yi-You Chiou

**Affiliations:** a Department of Radiology, Taipei Veterans General Hospital; b School of Medicine, National Yang-Ming University; c Department of Pathology and Laboratory Medicine, Taipei Veterans General Hospital, Taipei, Taiwan.

**Keywords:** angiography, computed tomography (CT), magnetic resonance imaging (MRI), pathology, ultrasonography, uncommon liver tumors

## Abstract

**Background:**

Beside hepatocellular carcinoma, metastasis, and cholangiocarcinoma, the imaging findings of other relatively uncommon hepatic lesions are less discussed in the literature. Imaging diagnosis of these lesions is a daily challenge. In this article, we review the imaging characteristics of these neoplasms.

**Methods:**

From January 2003 to December 2014, 4746 patients underwent liver biopsy or hepatic surgical resection in our hospital. We reviewed the pathological database retrospectively. Imaging of these lesions was reviewed.

**Results:**

Imaging findings of uncommon hepatic lesions vary. We discuss the typical imaging characteristics with literature review. Clinical and pathological correlations are also described. Primary hepatic lymphoma consists only of 1% of the extranodal non-Hodgkin lymphoma, and is defined as the one involving only the liver and perihepatic lymph nodes within 6 months after diagnosis. Combined hepatocellular and cholangiocarcinoma (cHCC-CC) shares some overlapping imaging characteristics with both HCC and cholangiocarcinoma because of being an admixture of them. Angiosarcoma is the most common hepatic mesenchymal tumor and is hypervascular in nature. Inflammatory pseudotumor is often heterogeneous on ultrasonography and with enhanced septations and rims in the portovenous phase after contrast medium. Angiomyolipoma (AML) typically presents with macroscopic fat components with low signal on fat-saturated magnetic resonance imaging (MRI) and presence of drainage vessels. Intraductal papillary neoplasm of the bile duct (IPNB) is thought of as a counterpart to the pancreatic intraductal papillary mucinous neoplasm. Most of the IPNBs secrete mucin and cause disproportional dilatation of the bile ducts. Mucinous cystic neoplasm (MCN) contains proteinaceous and colloidal components without ductal communication and characterizes with hyperintensity on T1-weighted imaging. Other extremely rare lesions, including epithelioid hemangioendothelioma and inflammatory pseudotumor-like follicular dendritic cell sarcoma, are also discussed. Hepatoblastoma and mesenchymal hamartoma, mostly in children, are also briefly reviewed as well.

**Conclusion:**

It is important for radiologists to be familiar with the typical imaging features of the uncommon hepatic neoplasms. If imaging findings are not typical or diagnostic, further biopsy is required.

## 1 Introduction

Imaging diagnosis of the hepatic tumors remains a daily clinical challenge. Among them, the most common primary malignant tumors are hepatocellular carcinoma (HCC) and cholangiocarcinoma. Metastasis is another common lesion as well. There are many literatures discussing these common hepatic lesions, and each of them has its own imaging characteristics. Beside the above tumors, other relatively less encountered lesions are often difficult to be diagnosed solely by imaging and are also less discussed. We review these uncommon hepatic lesions and describe the typical imaging findings with pathological correlation. Physicians should be familiar with these lesions to make a correct diagnosis.

From January 2003 to December 2014, 4746 patients underwent liver biopsy or hepatic surgical resection in our hospital. The most common hepatic neoplasms in our hospital were hepatocellular carcinomas (2176 cases, 45.8%), metastatic tumors (1490 cases, 31.4%), and cholangiocarcinomas (457 cases, 9.6%). The uncommon hepatic neoplasms include lymphomas, combined hepatocellular and cholangiocarcinomas, angiosarcomas, inflammatory pseudotumors, hepatoblastomas, angiomyolipomas, intraductal papillary neoplasms, mucinous cystic neoplasms, epithelioid hemangioendotheliomas, mesenchymal hamartomas, inflammatory pseudotumor-like follicular dendritic cell sarcoma, and hydatid cyst. We retrospectively reviewed the available images, including ultrasonography, computed tomography (CT), magnetic resonance imaging (MRI), and angiography images. After extensively reviewing the literature in English, the typical imaging findings, clues to differential diagnosis, clinical manifestations, and final pathologic correlation are discussed.

## 2 Methods

Ethical approval was waived for our study because the results for publication only involved deidentified imaging.

### 2.1 Data sources

We used the pathological database in our hospital. Retrospectively, the pathological diagnosis of the liver lesions from January 2003 to December 2014 was collected and reviewed. A total of 4746 patients underwent liver biopsy or surgical resection were included.

### 2.2 Setting

The most common pathologically diagnosed hepatic lesions in our hospital were hepatocellular carcinoma (2176 cases), metastatic tumors (1490 cases), and cholangiocarcinomas (457 cases). After exclusion of these common lesions, the relatively uncommon lesions were lymphomas (33 cases), combined hepatocellular and cholangiocarcinomas (26 cases), angiosarcomas (22 cases), inflammatory pseudotumors (22 cases), hepatoblastomas (13 cases), angiomyolipomas (13 cases), intraductal papillary neoplasms of the bile duct (11 cases), mucinous cystic neoplasms (8 cases), epithelioid hemangioendotheliomas (5 cases), mesenchymal hamartomas (2 cases), inflammatory pseudotumor-like follicular dendritic cell sarcoma (1 case), and hydatid cyst (1 case).

### 2.3 Radiologic review

Two board-qualified experienced gastrointestinal radiologists reviewed the provided images through our picture archiving and communication system (PACS). Ultrasonography, angiography, contrast-enhanced studies, including CT and MRI, were reviewed. Typical or specific images of each lesion were selected.

### 2.4 Pathological correlation

The pathological panels of the above selected cases were reviewed and confirmed by 1 pathologist after radiologic review.

## 3 Results

### 3.1 Patient group

After careful selection of the characteristic images and an extensive literature review, a total of 4746 cases were included. Those without definite pathological diagnosis and with more than 1 pathological diagnosis in the same lesion are excluded. The number of these cases is 466. Different gender compositions of the enrolled uncommon hepatic lesions in our collected cases are listed in Table 1 in detail. All listed lesions take up less than 1% of the pathological diagnosed hepatic lesions in our pathological database from January 2003 to December 2014. The case numbers and gender composition indicate the cases we reviewed and may not represent the true incidence.

**Table 1:**
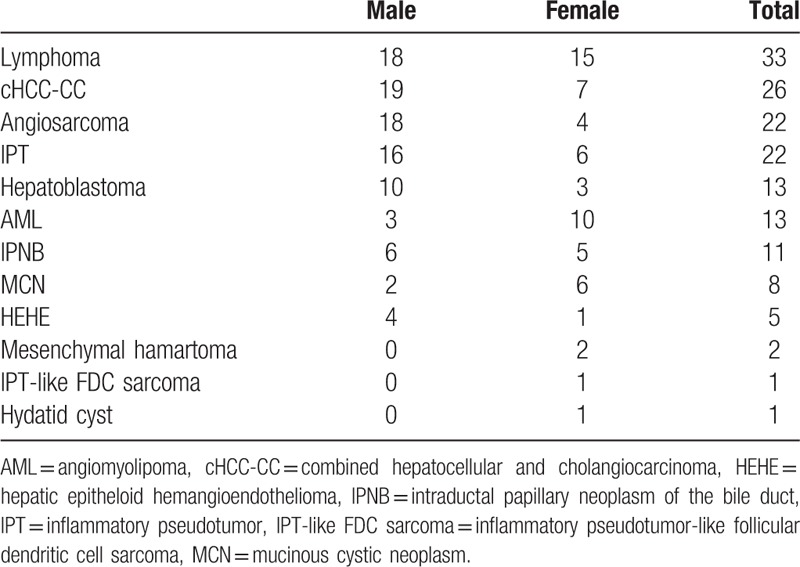
Cases enrolled in this observational study.

### 3.2 Imaging characteristics

The typical imaging findings and brief clinical manifestations are listed in Tables 2 and 3. The characteristic imaging and detailed discussion are as follows.

**Table 2:**
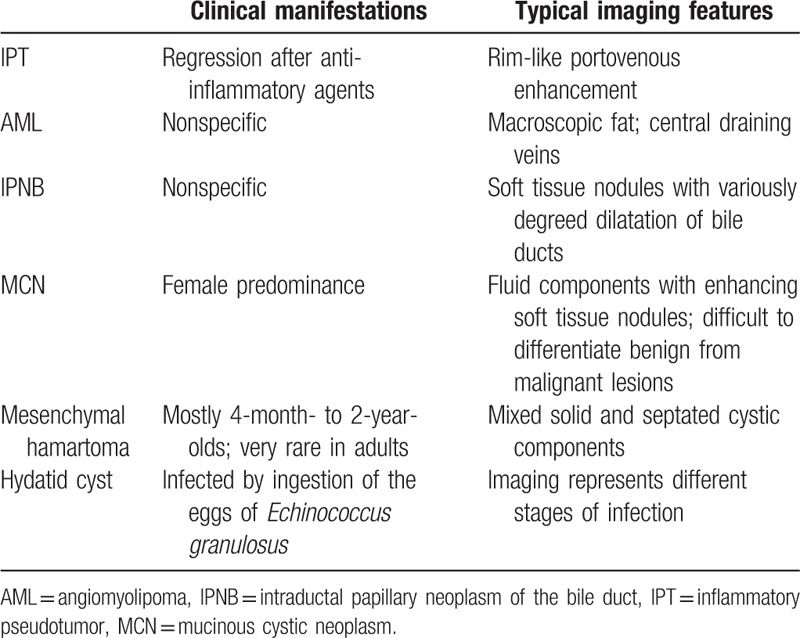
Characteristics of benign tumors.

**Table 3:**
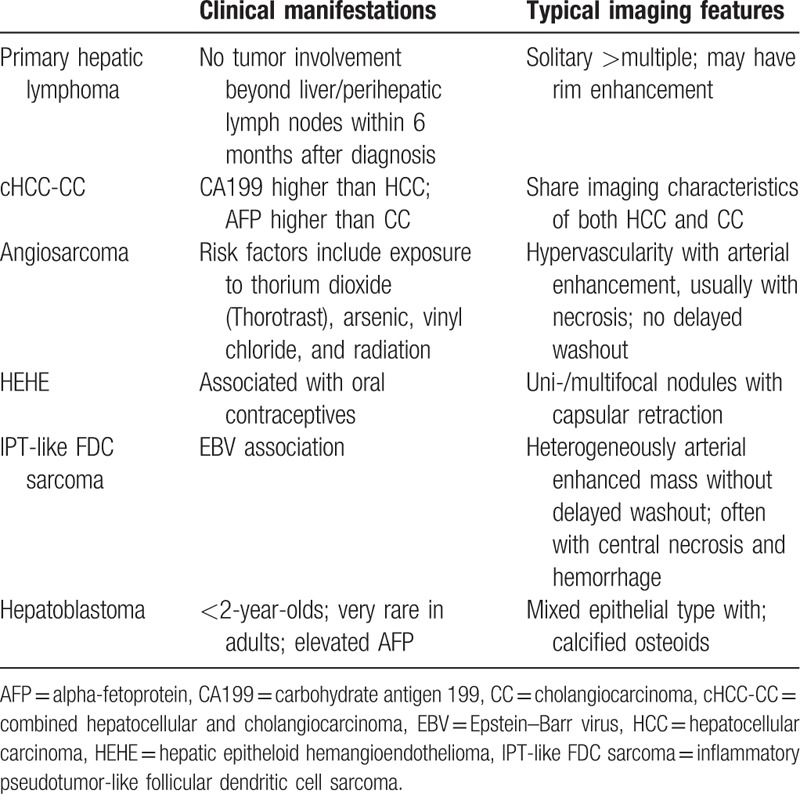
Characteristics of malignant tumors.

## 4 Discussion and review

### 4.1 Lymphoma

Hepatic lymphoma can be either primary or secondary. Overall, hepatic lymphoma contributes about 8% to the focal hepatic lesions.^[1]^

Secondary liver involvement of the lymphoma is common. The liver is the third-most common abdominal organ with lymphoma involvement, following the spleen and gastrointestinal tract.^[2]^ The lymphoma nodules often tend to be multifocal and with prominent lymphadenopathy not confined to perihepatic nodes. The most secondary hepatic lymphoma is non-Hodgkin lymphoma.^[3]^

Primary hepatic lymphoma (PHL), in contrast, constitutes only less than 1% of the extranodal non-Hodgkin lymphoma.^[4]^ PHL is defined as lymphoma involving only the liver and perihepatic lymph nodes without distant metastasis. A 6-month observation is often used in clinical settings for the distant metastasis after hepatic symptoms occur.^[5]^ Lei^[6]^ proposed that the clinical criteria of PHL exclude palpable lymph nodes, splenic, or bone marrow involvement. Many etiologic factors were described, yet the definite cause of the PHL remains uncertain. Hepatitis B virus (HBV), hepatitis C virus (HCV), the Epstein–Barr virus (EBV) in the posttransplant patients, and the human immunodeficiency virus (HIV) are statistically associated with the PHL.^[7]^ Gisbert et al^[8]^ reported that the prevalence of HCV infection in patients with B cell lymphoma (15%) is higher than in the general population (1.5%), suggesting a close association between HCV and B cell lymphoma. The symptoms of PHL are not specific, including typical systemic B symptoms or abdominal fullness.

**Figure 1:**
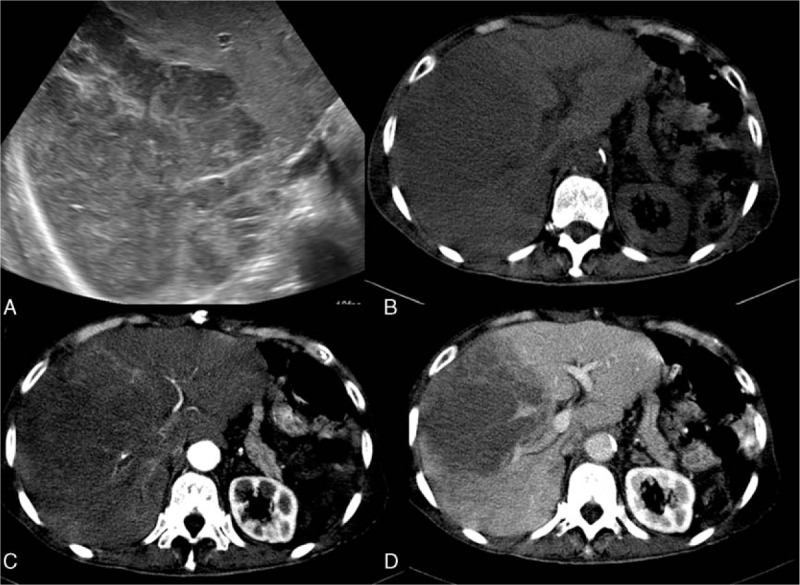
An 82-year-old man with suspicious primary lymphoma. No further involvement of other organs or marrow was noted 6 months after the diagnosis. A, Abdominal sonography shows a huge confluent hypoechoic mass at the right lobe of the liver. B, Precontrast CT. The mass shows relatively less enhancement than the surrounding liver parenchyma in both the arterial phase (C) and the delayed phase (D). Necrosis within the lesion is also depicted. CT = computed tomography.

The most frequent findings of lymphoma on ultrasonography are multiple focal liver lesions, hepatomegaly, splenomegaly, and lymphoadenopathies.^[2]^ The hepatic lesions may be either multiple hypoechoic lesions or a solitary confluent hypoechoic mass (Fig. 1A). PHL presents more commonly as a solitary mass, while secondary lymphoma tends to be multifocal. Color Doppler may be helpful in demonstrating the peripheral vascularity. On contrast-enhanced ultrasonography (CEUS), lymphomas tend to have a wash-out phenomenon in the late phase.^[9]^

Lymphoma typically presents as a relatively less enhanced lesion than the surrounding liver parenchyma in both the arterial and delayed phases on CT (Fig. 1). Lymphadenopathy may also be noted. The lymphadenomatous masses or nodules are in the soft tissue attenuation, and may contain hemorrhage or necrosis. T1 hypointensity and T2 hyperintensity are the typical imaging characteristics of lymphoma on MRI. These hypovascular lesions are with subtle enhancement, as seen on CT. The typical peripheral rim enhancement may be observed. Lymphadenomatous lesions present as high signals on DWI due to the relatively compacted cellular masses and high nuclear-to-cytoplasm ratio. The lower apparent diffusion coefficient (ADC) values of lymphoma were previously described.^[10]^

The major differential diagnoses of lymphoma are metastasis and infection. Hepatocellular carcinoma (HCC) typically enhances arterially and tends to wash out in the portovenous phases. Capsular retraction and enhancement may also be present in typical HCC; these imaging characteristics are not seen in lymphoma. Hypovascular metastasis, most commonly originating from the colon or lung, often manifests as multiple hypovascular nodules with rim enhancement, similar to lymphoma. Generally, metastasis is less likely in a cirrhotic liver. Clinical history may aid in the differential diagnosis and sometimes biopsy is needed. Both pyogenic abscesses and fungal microabscesses can appear similar to lymphoma, and may need further clinical clues for differential diagnosis.

PHL is treated with chemotherapy. Surgical resection is only preserved for selected patients with small and focal lesions. Since the most common entity of the PHL is diffuse large B-cell lymphoma, CHOP (cyclophosphamide, doxorubicin, vincristine, prednisone)-based chemotherapy is most commonly used and with relatively low risk of disease recurrence.^[4]^

### 4.2 Combined hepatocellular and cholangiocarcinoma

Combined hepatocellular and cholangiocarcinoma (cHCC-CC) is a rare entity. The incidence of this primary liver malignancy is about 0.4% to 14.2%.^[11]^ Allen and Lisa^[12]^ classified cHCC-CC into 3 categories. The first type is the separate type, also termed double cancer or collision tumor. The tumor consists of varying degrees of a combination of HCC and cholangiocarcinoma. Both the hepatocyte-derived and cholangiocyte-derived parts are separated. The second type is the combined type, or transition type, in which both HCC and cholangiocarcinoma grow contiguously yet independently. The third type is when both elements are almost indistinguishable from each other, termed mixed or intermediate type. Risk factors include cirrhosis, hepatitis B, hepatitis C, older age, and male gender.^[13]^

**Figure 2:**
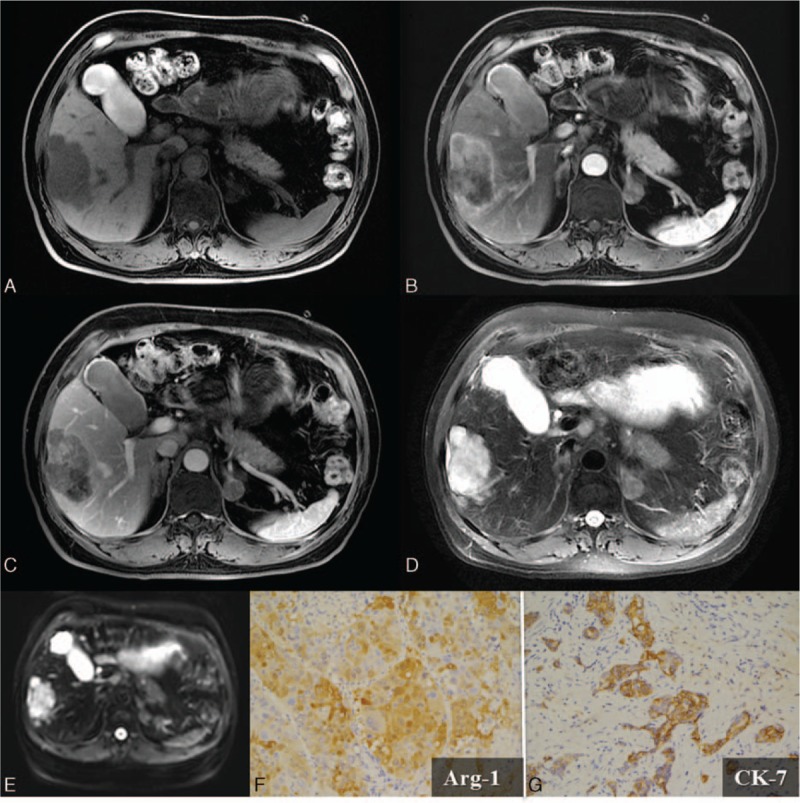
A 58-year-old man with a combined hepatocellular and cholangiocarcinoma (cHCC-CC). A, One 6.0 × 4.0 cm-sized mass in the right lobe of the liver is noted, which shows T1 hypointensity. Dynamic imaging studies reveal a typical arterial rim enhancement on panel (B) and centripetal enhancement in the delayed phase on panel (C). The tumor mass has a relatively irregular margin, which is a cHCC-CC characteristic. D, T2 hyperintensity of the lesion is depicted. E, Restricted diffusion is observed as high signal on DWI (*b* value = 800). Composition of both hepatocellular (Arginase-1; Arg-1; ×200) (F) and biliary (cytokeratin-7; CK-7; ×200) (G) differentiation was demonstrated by the immunohistochemical (IHC) stains. DWI = diffusion-weighted imaging.

The cHCC-CC shares some overlapping imaging characteristics with both HCC and cholangiocarcinoma because of being an admixture of them. The HCC component-dominant cHCC-CC tends to resemble HCC more, and vice versa. Typical cHCC-CC presents with arterial enhancement and capsular retraction on CT. The arterial enhancement of cHCC-CC tends to be at the periphery of the lesion, whereas typical HCC more commonly has homogeneous or heterogeneous arterial enhancement of the lesion as a whole. The target appearance of the arterial enhancement in cHCC-CC becomes washed out in the following portovenous or delayed phase. Centripetal enhancement of the central fibrous stroma and the dilatation of the bile duct may be present^[14]^ (Fig. 2). The imaging features on MRI are similar to those on CT. The cHCC-CC presents as a T2 hyperintense mass, typically with peripheral arterial enhancement and sometimes with centripetal enhancement in the portovenous phases. Intratumoral lipid may be observed in some cases. Nishie et al^[15]^ reported that the diagnostic rate of the cHCC-CC on enhanced CT was 33.3% for the overall 3 types of the tumor due to an atypical imaging appearance or different cell components. Without typical presentations, it may be difficult to completely exclude HCC or cholangiocarcinoma solely by imaging, and biopsy may be needed.

The major differential diagnosis of the cHCC-CC is the mass-forming cholangiocarcinoma. Both mass-forming cholangiocarcinoma and cHCC-CC have capsular retraction, bile duct dilatation, and peripheral rim enhancement. The shape of the former is more commonly lobulated due to the tendency to form satellite lesions when invading bile duct branches.^[16]^ In contrast, cHCC-CC tends to form an irregular shape due to its infiltrative nature.^[17]^ Some serologic markers, including higher serum levels of carbohydrate antigen-199 than in HCC and higher serum levels of alpha-fetoprotein (AFP) than in cholangiocarcinoma, have been described.^[18]^

Surgical resection is the treatment of choice when feasible. Yin et al^[13]^ reported that radical hepatic resection provides a better outcome for cHCC-CC. Reported prognosis varied. Most English literatures indicated poorer outcome of cHCC-CC, and some reported it as an intermediate prognosis between HCC and CC.^[14]^ Recurrent tumors of cHCC-CC are often hypovascular. Transarterial embolization is of limited role in the recurrent tumors, and radiofrequency ablation may be used. The use of chemotherapy remains controversial. Transplant is performed in selected patients, and the 5-year recurrence rate is 78%.^[14,16]^

### 4.3 Angiosarcoma

The primary angiosarcoma is the most common mesenchymal tumor in the liver, and makes up around 2% of the primary hepatic tumors.^[19]^ The most common primary locations of angiosarcoma are the skin and breast. The exact etiology of the tumor is uncertain, although several environmental exposures, including thorium dioxide (Thorotrast), arsenic, vinyl chloride, and radiation, have been reported as risk factors.^[20,21]^ The prognosis of angiosarcoma is poor, with the reported median survival of around 3.4 years.^[22]^ The clinical symptoms of angiosarcoma are nonspecific, such as abdominal pain, fatigue, or weakness. AFP is not elevated within most of the reported angiosarcomas. Surgical resection is usually performed only when the tumor is resectible. Adjuvant chemotherapy or radiation therapy is often applied, yet without significant survival benefits.^[23]^ Most angiosarcoma is not indicated for operation upon diagnosis, and palliative chemotherapy is then used. Transcatheter arterial chemoembolization (TACE) was been reported as one of the treatment options, regardless of intrahepatic metastasis.^[24]^

**Figure 3:**
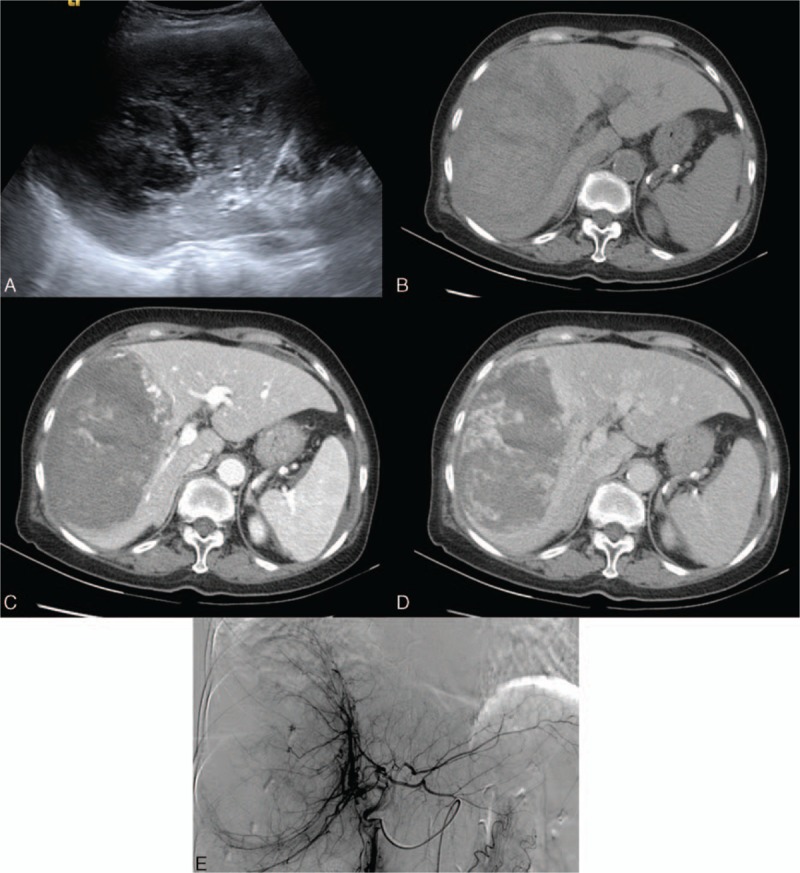
An 80-year-old woman with an angiosarcoma. A, A heterogeneously isoechoic mass with internal hypoechoic necrotic areas on sonography. B, The tumor mass has hypodense areas, indicating necrosis. C, A bizarrely shaped peripheral arterial enhancement is characteristic of angiosarcoma. D, Focally progressive but incomplete enhancement of the tumor is observed on the later phase. E, Hypervascularity with prominent vascular channels is depicted on celiac angiography.

The solid mass of angiosarcoma is composed of spindle cell-formed disorganized vessels, creating a sinusoidal or cavernous space. Therefore, this hypervascular tumor typically demonstrates irregular vascularity on enhanced images (Fig. 3). Spontaneous hemorrhage is a common complication, and emergent transarterial embolization is effective in controlling tumor bleeding. The angiosarcoma may present as multifocal or, less commonly, a solitary lesion. A heterogeneously hypoechoic to isoechoic mass, usually with some hypoechoic hemorrhage areas, is observed on ultrasonography (Fig. 3A). Vividly peripheral and nodular enhancement in the arterial phase on CEUS was previously described.^[25]^

Dynamic images depict the arterial enhancement of the angiosarcoma well on both CT and MRI. A hypodense mass with some internal necrotic areas is observed on the precontrast CT scan. Peripheral enhancement without significant washout in the portovenous phases is the typical imaging feature.^[26]^ Recent reports indicate that centripetal enhancement is actually rarely seen in angiosarcoma, which aids in the differential diagnosis of angiosarcoma from hemangioma.^[27]^ In fact, the focal arterial enhancing areas, with less enhancement than the aorta, bizarrely shaped peripheral enhancement, and the tendency to be multifocal lesions of the angiosarcoma, decrease the possibility of the hemangioma. Hypointense internal septations may be observed on T2-weighted images. Slight elevations of the ADC level were reported.^[28]^

### 4.4 Inflammatory pseudotumor

The inflammatory pseudotumor (IPT) is synonymous with the inflammatory myofibroblastic tumor and was first described by Pack and Baker.^[29]^ IPTs usually present with dominant spindle cell infiltrates and pathological IPTs usually present with various degrees of inflammatory cells. Tang et al^[30]^ reported that the IPTs are more common in young male adults, and often present with abdominal pain or fever; some cases are asymptomatic. The exact etiologies of the IPTs are uncertain. Risk factors, such as previous bile tract obstruction, infection, previous appendicitis, or underlying autoimmune disorders, have been described.^[31]^ Decreased tumor mass size, either spontaneously or after treatment with anti-inflammatory agents, was reported.^[30,32]^ The regression of the tumor mass after treatment with anti-inflammatory agents may increase the likelihood of IPTs. Generally, the prognosis of IPT is favorable, and there were rare recurrences after surgical resection.^[33]^

**Figure 4:**
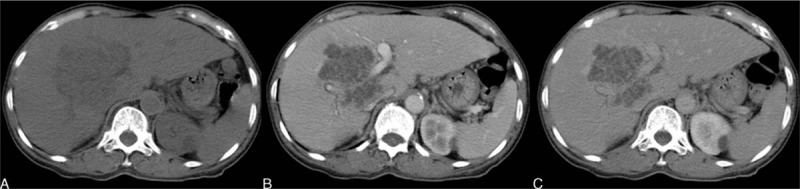
A 71-year-old woman with an inflammatory pseudotumor (IPT). A, Precontrast CT image reveals a relatively hypodense mass. B, Contrast injection revealed a lack of arterial enhancement in the tumor mass. C, Subtle later peripheral and septated enhancement in the delayed phase. CT = computed tomography.

The IPTs were well circumscribed, heterogeneous, and mixed with both echogenic and anechoic compartments in ultrasonography.^[34]^ Internal septations and calcification may be present. Ding et al^[35]^ described that IPTs had no enhancement in all phases after contrast injection in contrast-enhanced ultrasonography. The IPT masses typically show rim-like portovenous enhancement in CT imaging.^[32]^ An enhancement of the septations may also be observed. The various areas of delayed enhancement depend partially on the areas of fibrosis (Fig. 4).

On MRI, the IPTs typically present with hypointensity on T1-weighted images and isointensity to hyperintensity on T2-weighted images.^[36]^ The peripheral enhancement on the venous phases without significant arterial enhancement is concordant to that on CT.

As for the differential diagnosis, the major considerations are pyogenic abscess, metastasis, and peripheral cholangiocarcinoma. Patients with typical pyogenic abscesses might have infectious symptoms, such as leukocytosis or fever. Liquefied abscesses have typical central fluid-containing areas, which are different from the solid nature of IPTs. Peripheral cholangiocarcinoma might have the imaging features of a heterogeneous mass with delayed enhancement, and cannot be completely excluded solely by imaging. Kitajima et al^[37]^ postulated the use of diffusion-weighted imaging (DWI) with a low *b* value to differentiate IPT from cholangiocarcinoma because IPT demonstrates higher signal than cholangiocarcinoma. Some atypical IPTs may present with arterial enhancement, and are difficult to differentiate from hepatocellular carcinomas (HCCs). Since the imaging features of the IPTs share some overlapping characteristics with other primary hepatic malignancies, imaging diagnosis should only be made after the exclusion of these malignancies.

### 4.5 Hepatoblastoma

Heptoblastoma is the most common primary hepatic malignancy before 2 years of age. The peak incidence of the hepatoblastoma is around 6 months to 3 years.^[38]^ Adult hepatoblastoma has been reported but is extremely rare.^[39,40]^ Over half of the cases (68%) are diagnosed under the age of 2 and 90% of the cases are under 5 years of age. Male predominance has been reported. The clinical symptoms are usually abdominal fullness or jaundice. Elevated serum levels of AFP are seen in most cases. Ishak and Glunz^[41]^ classified the hepatoblastoma into epithelial, mixed epithelial, and mesenchymal types. By their names, the epithelial type is composed of fetal and embryonic cells and the mixed type is composed of mesenchymal and epithelial components as well.

**Figure 5:**
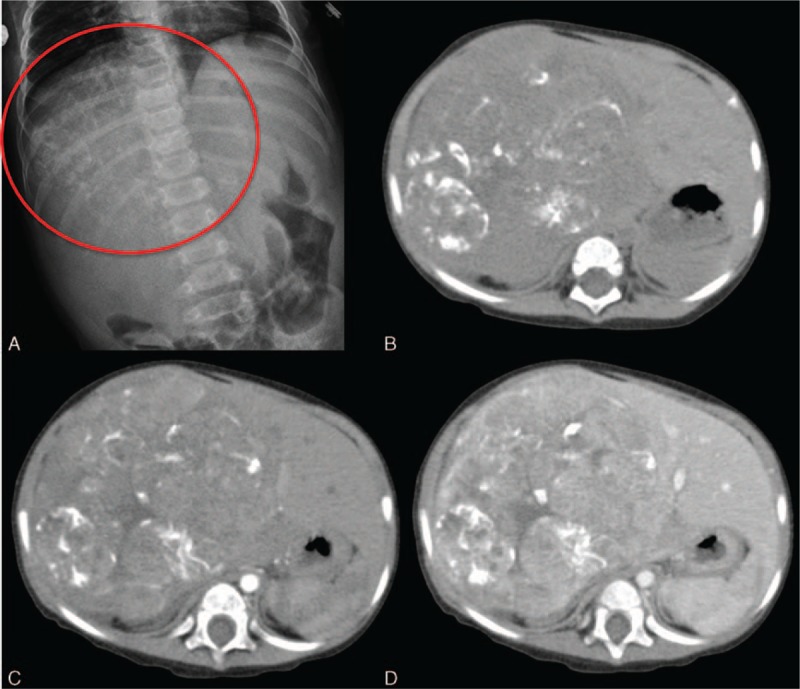
A 1-year-old girl with a mixed type hepatobastoma. A, Amorphous calcification (circle) is noted at the right upper abdomen on the plain radiograph. This characteristic should raise the suspicion of hepatoblastoma in the infant. B, Calcification within the tumor masses indicates osteoid, cartilaginous, or fibrous components, usually seen in mixed-type hepatoblastoma. C, D, Subtle arterial enhancement without significant washout on the later phases (D). Some subtle enhancement in the septations on the delayed phase is depicted as well.

The imaging features of the tumor depend on the various degrees of the epithelial or mesenchymal components. Epithelial-type hepatoblastoma is typically homogeneous, and the mixed type has a heterogeneous appearance due to the presence of osteoid, cartilaginous, or fibrous contents. Amorphous calcification may be observed in mixed-type hepatoblastoma (Fig. 5A and B). Subtle arterial enhancement with delayed enhancement in the septations is often noted on both CT and MRI (Fig. 5C and D). The use of ADC to assess treatment responses was reported.^[42]^

The major differential diagnosis of the hepatoblastoma is HCC. Both tumors cause AFP elevations and can have arterial enhancement. HCC is more commonly seen in a diseased liver after 5 years of age, whereas hepatoblastoma generally manifests in a cirrhotic liver before 5 years of age.^[43]^

Surgical resection is the treatment of choice. Yet, most of the hepatoblastomas are unresectible upon diagnosis. Neoadjuvant chemotherapy is applied to reduce the tumor size and most of them can then be treated with surgical resection. Systemic chemotherapy is used for those with disseminated presentation.

### 4.6 Angiomyolipoma

Hepatic angiomyolipoma (AML) is composed of 3 major components: thick-walled vessels (angio-), smooth muscle cells (myo-), and adipose cells (lipoma); the percentages of each component vary. Patients with tuberous sclerosis (TS) commonly develop renal AMLs, which have a tendency to hemorrhage. Hepatic AMLs, although uncommon, are also associated with TS complex.^[44]^ Furthermore, the presence of hepatic AML is related to the presence of renal AML, and is more common in patients with germline mutations in TSC2 (9q34) than in TSC1 (16p13).^[45]^ AML is a benign entity, and rare malignant transformation has been reported.^[46]^ Although invasive growth of the tumor can occur, this pathological finding is not diagnostic of malignancy.^[47]^ Most patients with hepatic AMLs present with no specific symptoms. There are no reliable serologic markers to date.

The typical hyperechoic lipomatous components may not be observed on ultrasonography. Therefore, hepatic AMLs are sometimes indistinguishable from common hemangiomas. Hepatic AMLs present with an inhomogeneous and hyperenhancing pattern in the arterial phase and prolonged enhancement in the later phases. Identification of the tumoral efferent veins toward the hepatic vein makes the impression more likely to be an AML.^[48]^ The angiomyomatous components of AMLs demonstrate arterial enhancement and the lipomatous components have with hypodense lipid attenuation without significant enhancement on CT. Overall, a heterogeneous enhanced mass is often observed. The degrees of fat components vary from 10% to 90%, and a cutoff value of minus 20 Hounsfield units (HU) by CT was used to determine the definite presence of adipose tissues.^[49]^

**Figure 6:**
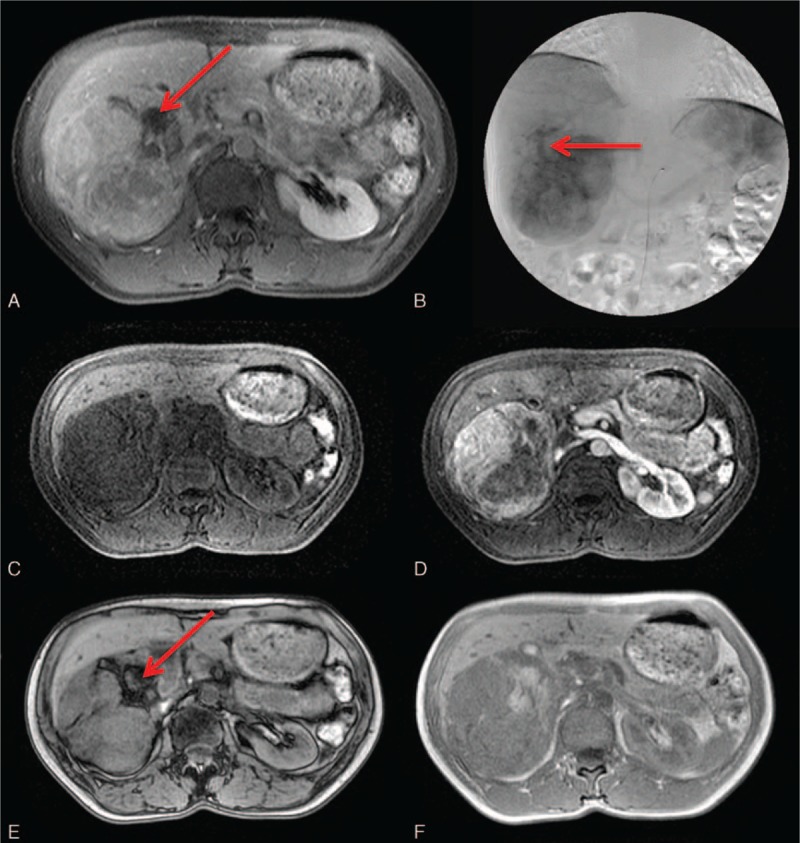
A 40-year-old woman with a hepatic angiomyolipoma (AML). No significant discomfort was noted. A, The presence of macroscopic fat is clearly detected as low signal (arrow) by T1-weighted MRI with fat suppression. B, Angiography shows a draining vein (arrow) from the center of the tumor mass; this is a key characteristic of AML. Pre- (C) and post- (D) contrast T1-weighted images depict the inhomogeneous enhancement due to various degrees of angiomyomatous contents. On dual gradient-echo images, a chemical shift artifact is observed in the cancellation of the signal (arrow) on the out-of-phase (E), compared with the in-phase image (F). MRI = magnetic resonance imaging.

The macroscopic fat components present with low signal in fat-suppression images on MRI (Fig. 6). Although less common, cancellation of the signal may sometimes be observed in out-of-phase dual gradient-echo images as well. Fat components demonstrate hyperintensity on T1-weighted images if fat components are abundant. Dynamic images show a tumor mass with arterial enhancement and prolonged enhancement in the later phases (Fig. 6C and D). The central draining vein may be depicted in the portovenous phase, and identification of the central vessels of the AML is crucial in differential diagnosis (Fig. 6B). This typical characteristic excludes the fat-containing HCC and focal nodular hyperplasia, which tend to have vessels in the periphery.^[50]^

### 4.7 Intraductal papillary neoplasm of the bile duct

Intraductal papillary neoplasm of the bile duct (IPNB) has been a distinctive part of biliary lesions in the WHO classification since 2010.^[51]^ The previously termed cystadenomas/cystadenocarcinomas with bile duct communication are now classified as IPNBs. Tumors with intraductal growth of the papillary fronds and fine vascular cores, such as some intraductal cholangiocarcinomas and papillary carcinomas, are also currently considered IPNBs. An IPNB is defined as a premalignant lesion and is subgrouped into intraductal papillary neoplasms (IPNs) with low-, intermediate-, or high-grade intraepithelial neoplasia and IPNs with invasive carcinoma. An oncogenic pathway of adenoma-carcinoma with KRAS activation and a loss of function of the tumor-suppressor genes TP53 and p16 has been postulated.^[52]^ Long-term survival may be achieved after complete resection.^[53]^ The IPNB is thought of as a counterpart to the pancreatic intraductal papillary mucinous neoplasm.^[54]^

IPNB occurs as either intrahepatic or extrahepatic neoplasms. The papillary or villous growth of the tumors in the bile ducts vary in degrees. Most of them secrete viscous mucin, causing bile duct dilatation. Lim et al^[55]^ reported different types of IPNB-related bile duct dilatation, including generalized, segmental and aneurysmal appearances. The disproportional bile duct dilatation of the aneurysmal or cyst-like IPNB refers to insufficient downstream biliary outflow due to the viscous nature of the mucinous secretion.^[54]^

**Figure 7:**
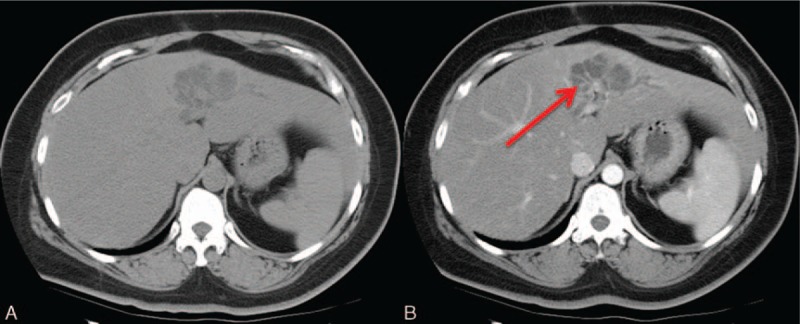
A 42-year-old woman with an intraductal papillary neoplasm of the biliary duct (IPNB). A, Focal aneurysmal, or cystic-like dilatation of the biliary ducts. The disproportionate dilatation the ducts is due to the high viscosity of the secreted mucin. B, A tiny, yet visible, enhanced intraductal soft tissue nodule is observed (*arrow*).

The imaging characteristics of IPNBs are soft tissue-attenuated tumor masses and variously degreed dilatations of the bile ducts (Fig. 7). Ultrasonography, CT, and MRI all demonstrate the above features well. On dynamic CT, a typical IPNB is relatively hyperdense or isodense to the surrounding parenchyma in the arterial phase and without hyperdensity in the portovenous phase.^[56]^ Both CT and MRI have high sensitivities in the detection of the intraductal masses, and the use of MR cholangiography (MRCP) further depicts the dilatation of the bile ducts and the extent of ductal infiltration well. DWI may be beneficial in tumor detection and to determine its invasiveness.^[57]^

Although IPNB is a disease spectrum ranging from benign lesion to malignancy, management based on this histological spectrum is not yet determined. Overall, a long-term survival may be achieved with complete resection.^[53]^

### 4.8 Mucinous cystic neoplasm

Mucinous cystic neoplasm (MCN) of the liver, or previously known as cystadenoma, is one of the biliary benign entities. According to the WHO classification in 2010, mucinous cystic neoplasm is defined as a cystic lesion with epithelial origin in association with ovarian-type subepithelial stroma.^[51]^ Cystadenoma and its malignant counterpart, cystadenocarcinoma, should be better termed “MCN with low-, intermediate-, or high-grade dysplasia” and “MCN with an associated invasive carcinoma,” as suggested by the latest edition of the WHO classification.^[51]^

The MCN is predominantly in females. The tumor contains variable degrees of proteinaceous and colloidal contents. Septations and compartments are typically present. Although malignant transformation exists, the presence of soft tissue attenuation does not always indicate malignancy. Its malignant counterpart, previously termed cystadenocarcinoma, may not be fully excluded solely by imaging.

**Figure 8:**
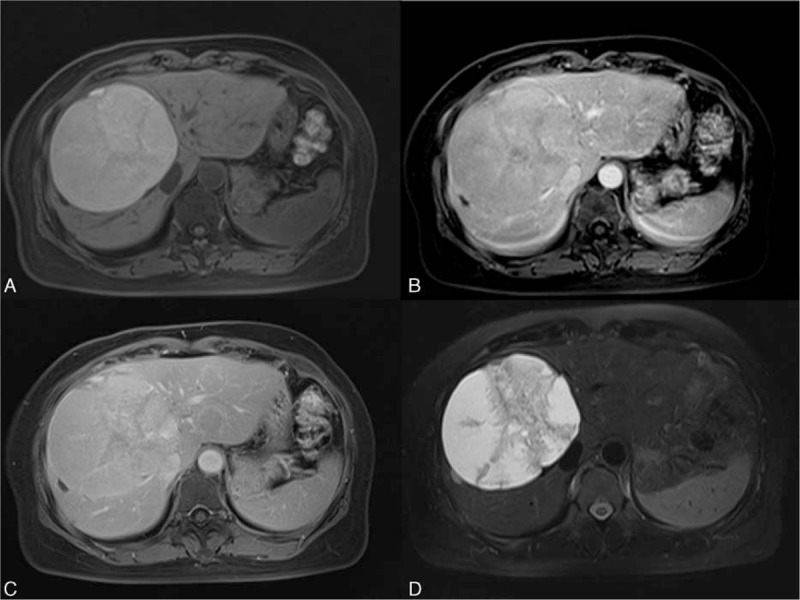
A 50-year-old woman with a mucinous cystic neoplasm (MCN). MCN with low-grade dysplasia was diagnosed after surgical resection. A, T1 precontrast image shows a hyperintense mass with some internal septation at the right lobe of the liver. The T1 high signal is due to high proteinaceous and colloid contents. The fluid-containing parts show no enhancement after contrast on T1-weighted images in the arterial (B) and the later phases (C). D, T2-weighted image with fat suppression depicts the fluid-containing parts with high signal. Note the septations are also clearly identified on T2WI.

The imaging characteristics of the MCN include a well-defined margin with septations, hypodense fluid-containing components, and rare enhancement except for the mural nodules. Hemorrhage or calcification may be present. On MRI, T1 hyperintensity is observed due to its proteinaceous and colloidal contents (Fig. 8).

The differential diagnosis of the MCN with other benign cystic lesions is important. Other benign cystic lesions, such as simple cyst or biliary hamartoma, are sometimes treated by drainage to relieve the mass effect, whereas MCN needs resection to prevent recurrences.^[58]^ The purely homogeneous appearance of the simple cyst and hamartoma can lead to the correct diagnosis. Zen et al^[59]^ reported that the differential diagnosis between IPNB and MCN is important due to their different natures; IPNB tends to grow in connection with the bile duct, whereas MCN does not. MRCP depicts the relationship of the lesion to the bile ducts well and therefore aids in diagnosis.

### 4.9 Epitheloid hemangioendothelioma

Hepatic epitheloid hemangioendothelioma (HEHE) is a rare entity of vascular origin, with an incidence less than 0.1 per 100,000 people per year. It is a low-grade malignant tumor that has an intermediate clinical outcome between benign hepatic cavernous hemangioma and malignant angiosarcoma.^[60]^ The peak incidence is at ages between 30 and 50 years old, and the disease more commonly affects females.^[61]^ It has been proposed that taking oral contraceptives or having contact history with vinyl chloride may increase the risk of HEHE.^[61]^ The most common clinical presentation of HEHE is abdominal pain, especially the right upper quadrant of the abdomen. About one-fourth of patients were asymptomatic initially. Hepatomegaly and weight loss are also common initial presentations. Lab data are not helpful in diagnosing HEHE. Alkaline phosphatase (Alk-P) may be elevated in some patients with HEHE, though it is a nonspecific marker.^[62]^

**Figure 9:**
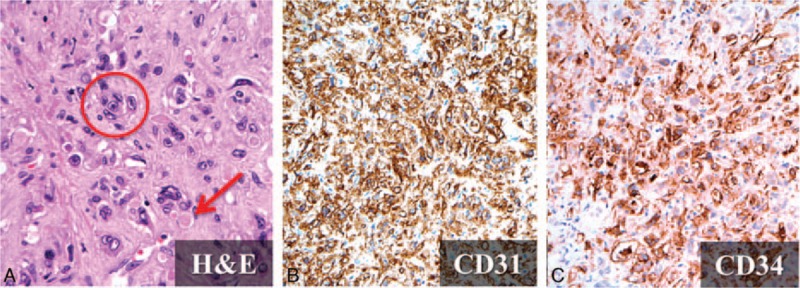
Histopathology of hepatic epitheloid hemangioendothelioma (HEHE). A, Microvascular channel with red blood cells within the lumens (*arrow*) and the presence of high nuclear-cytoplasmic ratio atypical cells (*circle*) are depicted after hematoxylin and eosin (H&E) staining (×400). CD31 (×200) (B) and CD34 (×200) (C). The negative epithelial markers are not shown.

HEHE is diagnosed by pathologic examination. The histopathologic features of HEHE include mixed epithelioid and dendritic cells in a proliferative fibrous stromal background. Endothelial cells are stained positively by immunostaining markers, including antibodies for factor VIII-related antigen, CD31, or CD34 (Fig. 9). The epithelial markers should stain negatively.^[63]^

**Figure 10:**
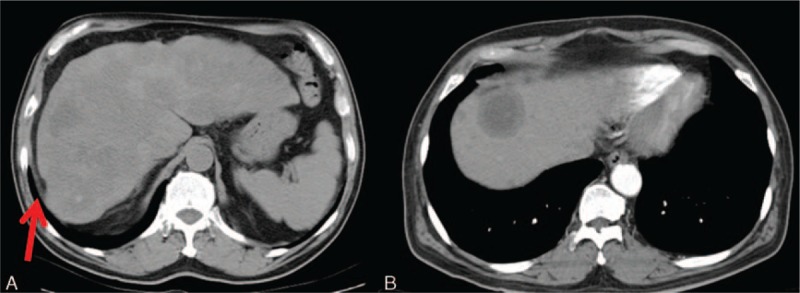
Two other cases of HEHE. A, Multifocal hepatic tumors with capsular retraction (*arrow*). B, Peripheral rim demonstrates a “target-like” appearance.

Typical image findings of HEHE include unifocal or multifocal nodules in the liver, with a predilection for locations such as the right lobe of the liver and the subcapsular regions. Capsular retraction is a common finding.^[64]^ In the literature, multifocal involvement (multifocal nodular and diffuse coalescent) (85%) is more common than unifocal involvement^[62]^ (Fig. 10). The right lobe of the liver is more affected than the left lobe in both forms of HEHE. Hypertrophic changes may be observed in the uninvolved lobe of the liver.^[60]^ HEHE is hypoattenuated and calcification foci can be seen in about 20% patients on precontrast CT.^[65]^ HEHE typically demonstrates hypointensity on T1-weighted images and heterogeneous hyperintensity on T2-weighted images, compared with the normal liver parenchyma, on MRI.^[64]^ There are diversities of enhancing patterns on both postcontrast CT and MR studies, which include peripheral or target enhancement, with central hypoenhancement. HEHE occasionally may show a peripheral hypodense or hypointense rim, which correlates with the avascular rim seen under pathologic examination.^[60]^ Alomari^[66]^ reported a characteristic “lollipop sign” for HEHE, which is comprised of a hepatic or portal vein tapering at the periphery of the tumor, with the central avascular core identified on CT or MRI. The prognosis of HEHE depends on the presence of extrahepatic involvement at the time of diagnosis. The most common sites of extrahepatic involvement include the lungs, lymph nodes, peritoneum, omentum, and bones.

Surgical resection and liver transplantation are considered the treatments of choice. Liver transplantation is used in patients with multiple tumors and extensive hepatic involvement. The roles of radiation and chemotherapy are still undetermined.

### 4.10 Mesenchymal hamartoma

The mesenchymal hamartoma is the second-most common hepatic benign tumor in children, following infantile hemangioendothelioma. The peak incidence of the tumor is between 4 months to 2 years of age, and very rarely seen in adults.^[67]^ The mesenchymal hamartoma is composed of mesenchyme, bile ducts, and hepatocytes, each to various degrees. The loosely arranged mesenchyme has hepatocytes lining the sides, and the voiding spaces are often filled with fluid. The presence of fluid is probably due to cystic-degenerated mesenchyme, lymphatic, or bile duct obstruction. Elevation of the AFP level was noted in some cases.^[68]^

**Figure 11:**
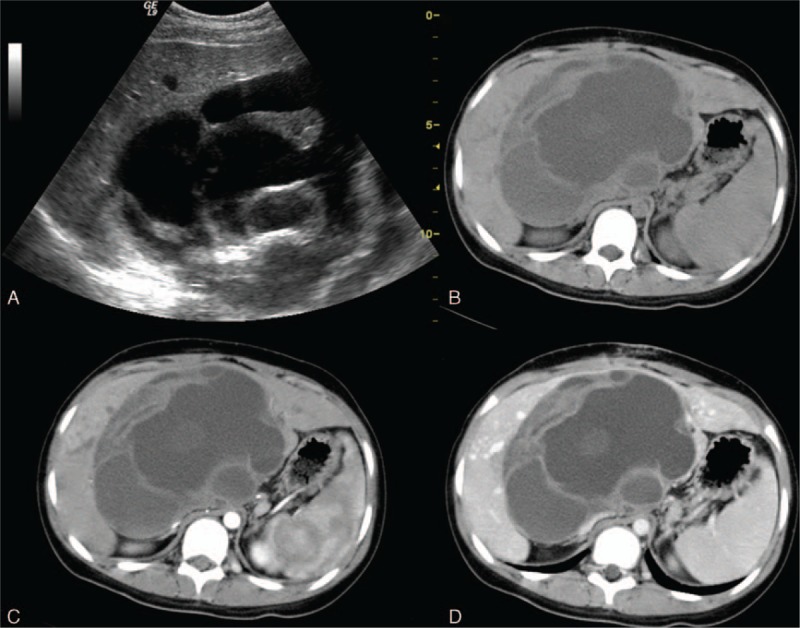
An 11-year-old girl with a mesenchymal hamartoma who presented with abdominal fullness. A, Combined cystic and solid mass is depicted on sonography. Dynamic CT with precontrast (B), arterial phase (C), portovenous phase (D) shows progressive enhancement of the septated and stromal parts of the tumor. The cystic part is without enhancement in all phases.

Being an admixture of mesenchyme, bile ducts, and hepatocytes, the mesenchymal hamartoma has both cystic and solid components. Rarely, the tumor may contain only the solid part, which makes it difficult to differentiate it from other solid tumors in children, such as hepatoblastoma. Typically, the tumor appears as a well-defined mass, with cystic components and internal septations. The cystic components may be either homogeneous or heterogeneous, depending on the proteinaceous content. Slight and delayed enhancement of the septations is observed (Fig. 11). Calcification may be noted, yet is relatively uncommon. The soft tissue components may present hypointensity on both T1- and T2-weighted images due to their fibrotic septatic nature. Cystic components generally express hyperintensity on T2-weighted images, and various intensities on T1-weighted images for the possible presence of proteinaceous contents and the stromal elements.

Most of the mesenchymal hamartomas can increase their sizes in the first several months. Surgical resection is the definite treatment option. Spontaneous regression has been reported in some literatures.^[67,68]^

### 4.11 Inflammatory pseudotumor-like follicular dendritic cell sarcoma

The inflammatory pseudotumor-like follicular dendritic cell (IPT-like FDC) sarcoma is an uncommon malignancy; the rare entity was first described by Selves et al^[69]^ in 1996. It is a distinctive clinicopathologic variant, instead of simply a morphologic variant, of the conventional FDC sarcoma. Compared with the conventional FDC sarcoma, which lacks gender predilection and takes place in nodal and various ranges of extranodal sites, IPT-like FDC sarcoma occurs almost exclusively in the liver or spleen and occurs predominantly in females.^[70]^ Due to the confined locations of the tumor mass, the disease extent is relatively indolent in IPT-like FDC sarcoma.^[71]^ A nearly 100% association between Epstein–Barr virus (EBV) and IPT-like FDC sarcoma was reported. Surgical resection of the tumor is the standard treatment; the use of adjuvant chemotherapy remains controversial.^[72]^

**Figure 12:**
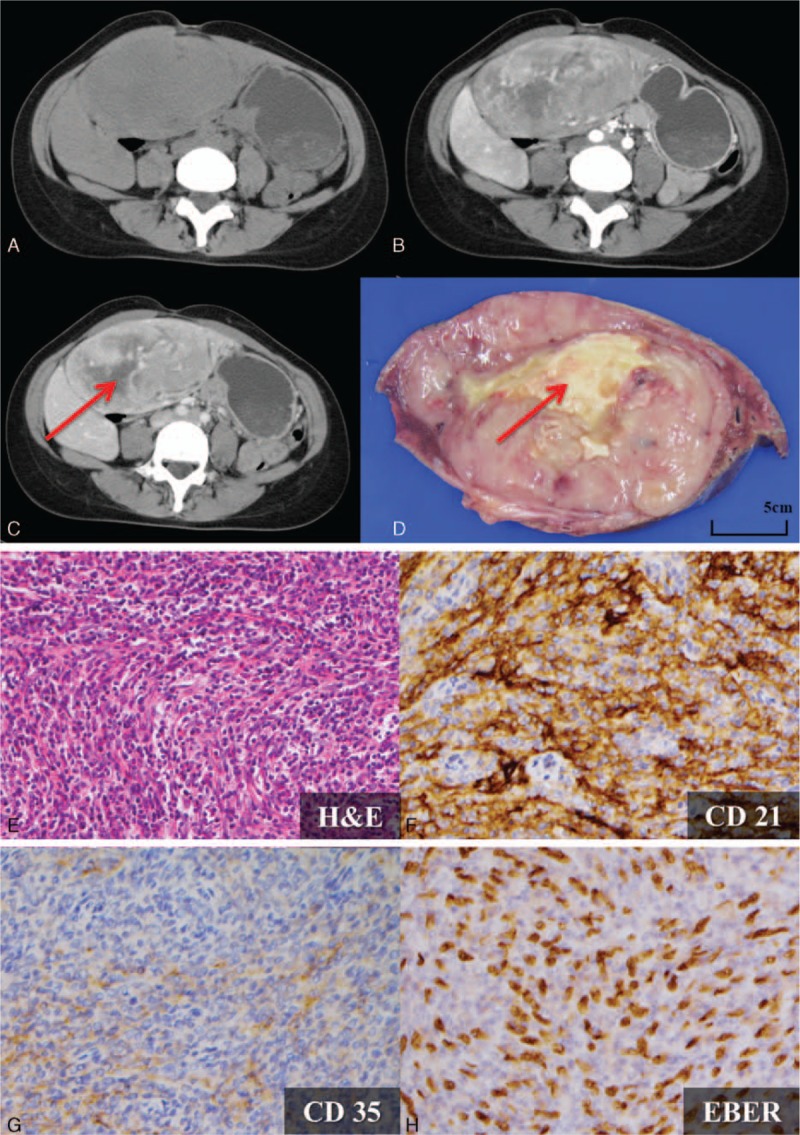
A 31-year-old woman with an inflammatory pseudotumor-like follicular dendritic cell (IPT-like FDC) sarcoma presented with a palpable abdominal mass. Tumor markers, including alpha-feto protein (AFP), CEA, CA125, CA153, and CA199, were all within normal limits. HBsAntigen, HBeAntigen, anti-HBe antibody, anti-HBc IgG, and anti-HCV antibody were nonreactive. A–C, One well-circumscribed mass with heterogeneous arterial enhancement (B) and without significant portovenous washout (C) was observed. Central necrosis is also noted (*arrow*). D, Gross finding after segmentectomy. Central necrosis (*arrow*) is observed, corresponding to the preoperative imaging findings. E, H&E staining (×200) revealed scattered spindle-shaped cells admixed with abundant lymphocytes and plasma cells in the background. Positive FDC markers with (F) positive CD21 (×400) and (G) focally positive CD 35 (×400) are shown. H, Positive Epstein–Barr virus-encoded small RNAs (EBER; ×400) indicate the strong relation with the EBV virus. EBV = Epstein–Barr virus.

A definite diagnosis of IPT-like FDC sarcoma is challenging, due to lack of reliable serologic markers, nonspecific symptoms, and nonspecific imaging features. The tumor appears as a well-defined, heterogeneous arterial enhanced mass without significant portovenous washout. Central necrosis or hemorrhage may be present (Fig. 12A–D). These features may overlap with those in other hepatic malignancies. Tissue biopsy is suggested when IPT-like sarcoma is considered.

The definite diagnosis of IPT-like sarcoma relies on histopathology. The tumor is a mixture of lymphocytes, plasma cells, and spindle cells. Positive staining with at least one of the FDC markers (CD21, CD35, CD23, or CNA42) should be fulfilled. All tumor cells show strong nuclear in situ labeling for EBV-encoded small RNAs (EBER), indicating a strong relation to EBV^[72]^ (Fig. 12E–H).

### 4.12 Hydatid cyst

The presence of a hydatid cyst is due to the infection of *Echinococcus* tape worms. Human beings usually become infected by eating the eggs from contaminated food. The embryos then invade the bowel mucosa, and enter the liver parenchyma through portovenous flow.^[73]^

**Figure 13:**
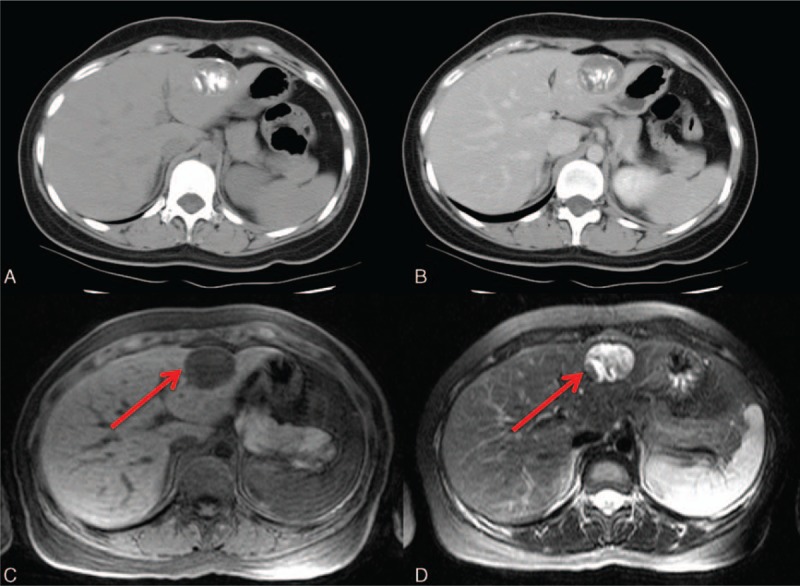
A 24-year-old woman with Echinococcus infection. A, Partially calcified hydatid cyst is well depicted on the precontrast CT image. The calcification is a consequence of the host–antigen reaction. B, The cystic component is without enhancement. C, Typical hypointensity on the T1-weighted image. D, Typical hyperintensity on the T2-weighted image. Note the characteristic hypointense rim on both the T1- and T2-weighted images (*arrows* in (C) and (D)), indicating the collagen produced by the host. This is the “frontline” of the host and the antigen.

The imaging appearances of the hydatid cyst range from unilocular-cystic to multilocular-cystic and partially calcified to completely calcified (Fig. 13), depending on the stages of the parasite infection. Cystic calcification is usually on the periphery. When complete calcification of the lesion is observed, the parasite is nearly dead. When unilocular, the hydatid cyst may resemble a simple hepatic cyst. DWI may be a useful tool for the differential diagnosis because a hydatid cyst presents with high signal, whereas a simple hepatic cyst does not.^[74]^ Intrabiliary rupture of the hydatid cyst can occur, and MRCP may help detect the connection of the lesion to the bile duct.^[75]^

A hypointense rim on both T1- and T2-weighted images may be observed on MRI, which indicates the collagen produced by the host on the periphery of the pericyst. This thin hypointense rim is recognized as the “frontline” between the host and the parasite antigen.^[74]^ Collapsed parasite membranes may also be noted as linear structures within the lesion. The hydatid matrix is typically a hypointensity in T1-weighted images and a hyperintensity in T2-weighted images (Fig. 13C and D).

## 5 Conclusion

It is important for radiologists to be familiar with the typical imaging features of the uncommon hepatic neoplasms. If imaging findings are not typical or diagnostic, further biopsy is required.
